# On the Penrose and Taylor–Socolar hexagonal tilings

**DOI:** 10.1107/S2053273317003576

**Published:** 2017-05-07

**Authors:** Jeong-Yup Lee, Robert V. Moody

**Affiliations:** a Department of Mathematics Education, Catholic Kwandong University, Gangneung, Gyeonggi-do, 210-701, Korea; b Department of Mathematics and Statistics, University of Victoria, Victoria, British Columbia V8W 3P4, Canada

**Keywords:** Penrose tiling, Taylor–Socolar tiling, double hexagon tiling, nested triangularizations, inverse sequences of hierarchical lattices

## Abstract

The intimate relationship between the Penrose and the Taylor–Socolar tilings is studied, within both the context of double hexagon tiles and the algebraic context of hierarchical inverse sequences of triangular lattices. This unified approach produces both types of tilings together, clarifies their relationship and offers straightforward proofs of their basic properties.

## Abstract 2

A uniform geometric/algebraic approach to the Penrose and Taylor–Socolar hexagonal tilings is given, which clarifies their construction and the way in which they are inter-related.

## 1. Introduction   

From the very beginning, aperiodic tilings have played a significant role in unraveling the mysteries of aperiodic crystals. Knowing what is mathematically possible has often turned out to be a crucial element in conceiving what might be physically realizable. In this paper we discuss two remarkable aperiodic tilings of the plane that are built out of one of the most basic of all crystallographic structures: the standard periodic hexagonal lattice.

Also, from the very beginning, there arose the question of what might be the minimum number of different prototiles necessary for a system of tiles and corresponding matching rules that permit, and only permit, aperiodic tilings. The very first aperiodic tilings involved thousands of prototiles. The famous aperiodic tilings of the plane like the rhombic Penrose and the Ammann–Beenker tilings are each based on just two prototiles, the allowable motions being translations and rotations. This of course immediately raises the question of whether aperiodic tilings based on just one prototile are possible.

**Figure 1:**
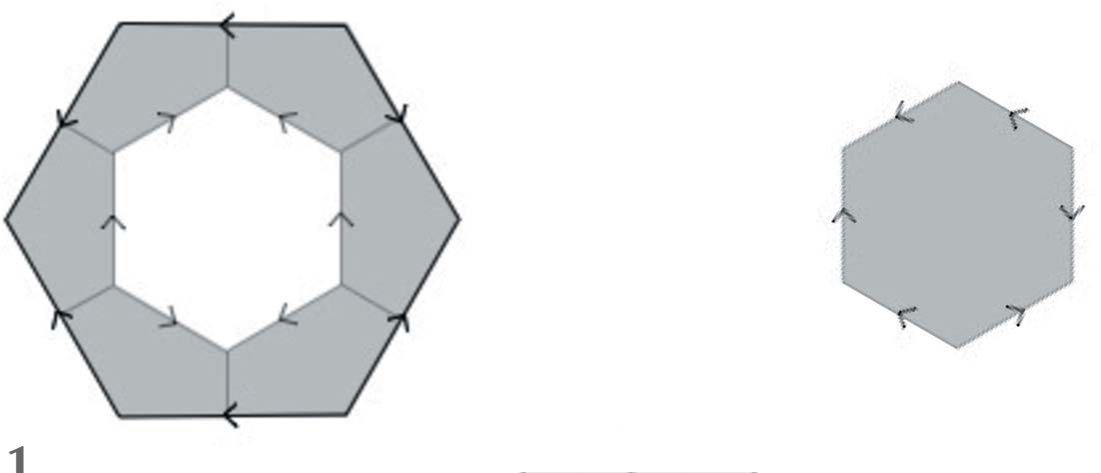
A well arrowed hexagon is shown on the right. It has one pair of opposite sides whose arrows face in the same direction, thus providing an orientation for the tile. A double hexagon tile is on the left, the key feature being that the orientations of the inner and outer arrowed hexagons are at right angles. When three double hexagon tiles meet at a vertex, the gray parts around the vertex form corner hexagons (see Fig. 2 ▸). The assumption of *legality* allows completion of the arrowing on the corner hexagons to well arrowed hexagons, as indicated on the right.

Taylor (2010 ▸) and Socolar & Taylor (2011 ▸) introduced a planar aperiodic tiling which can be built from a single hexagonal prototile allowing translations, rotations and *reflections*. The tiling is based on the familiar hexagonal tiling of the plane, but if one distinguishes the prototile in its direct and reflected forms, then the matching rules allow only aperiodic tilings to appear. Their work revived interest in the much earlier work of R. Penrose. In Penrose (1997 ▸), he had introduced an aperiodic tiling, also based on marked hexagonal tiles, but additionally involving two other types of thin edge tiles and small corner tiles, which he called a 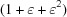 tiling. However, already in this paper he had introduced arrowed double hexagon tiles as an alternative way to represent the tiling (see Fig. 1 ▸). Later, in his online notes (Penrose, 2010 ▸) he expanded upon the double tile theme and pointed out the essential matching rules that make them work.

In both the Taylor–Socolar and Penrose cases, the ‘matching rules’ somewhat stretch the original notion of matching rules, so there can remain some controversy about whether these are strictly aperiodic monotiles. However, that is not an issue here. These tilings are interesting, and puzzling too, for although the Taylor–Socolar hexagonal tilings, henceforth called Taylor–Socolar tilings or T–S tilings, and the Penrose hexagonal tilings (Penrose tilings^1^) seem deeply related, that relationship is somewhat obscure. The two tilings are not mutually locally derivable (MLD) (Baake *et al.*, 2012 ▸; Baake & Grimm, 2013 ▸) in the technical sense, but are mutually derivable in a rather different sense that we shall explain.

In Lee & Moody (2013 ▸) we put forward a development of the T–S tilings based on the underlying hierarchical system of nested equilateral triangles that are so prominent in both the T–S tilings and the Penrose tilings. The aperiodicity of the tilings comes from this hierarchical structure, and indeed these tilings seem to have been invented with precisely this feature in mind. The structure of nested triangles has an algebraic interpretation as an inverse system of finite groups, arising from the standard triangular lattice and its natural triangular sublattices, and is closely related to the 2-adic integers. In Lee & Moody (2013 ▸) we made this algebraic interpretation the basis out of which we constructed the T–S tilings. In fact, as long as the nested system of triangles is generic, meaning that it is free of singularities (like points which are simultaneously the vertices of triangles of unbounded size), then there is a unique T–S tiling belonging to it. We shall see that the same type of mathematics applies to the Penrose tilings, and not surprisingly the two inverse systems are deeply connected.

A more detached look at the double hexagon tilings reveals that they actually incorporate both types of tilings simultaneously. This association is not entirely new (Baake *et al.*, 2012 ▸; Baake & Grimm, 2013 ▸), but in this paper it is the double hexagon tilings that are taken as the fundamental objects and they serve as the parents of the two individual types (Penrose and T–S) of hexagonal tilings. Thus, we may think of the two tilings as siblings of each other. Algebraically, a double hexagon tiling corresponds to a matched pair of inverse sequences, and with these we can see how the algebra and geometry fit seamlessly to elucidate each other. The paper offers a unified treatment of the two tilings along with proofs of the implied hierarchical structuring and the aperiodicity.

## 2. Double hexagon tiles and their tilings   

An arrowed hexagon is a regular hexagon in which each side has been given a direction, indicated by an arrowhead. An arrowed hexagon is called *well arrowed* if, up to rotation, the arrows form the pattern shown on the right side of Fig. 1 ▸. In fact, all three hexagons in this figure are well arrowed. The structure of the well arrowed hexagon gives it a well defined orientation in the plane, namely that provided by the two parallel arrows facing in the same direction.

If we start at any edge of a well arrowed hexagon and then look at every alternate edge as we go around it, we notice that arrows on the three edges always are a mixed type of clockwise and counter-clockwise. We notice also the useful fact that if we have a hexagon and three alternate edges have been arrowed so as to be of mixed type, then there is a unique way to complete the arrowing to make it into a well arrowed hexagon.

A hexagonal tiling of the plane with well arrowed hexagons is called *well matched* if the hexagons meet edge to edge and the arrows of these coinciding edges also coincide – that is, they point in the same direction. We are only interested in well matched tilings of arrowed hexagons.

A double hexagon tile (or double hex tile) consists of a pair of well arrowed hexagons, one within the other, as shown on the left side of Fig. 1 ▸. The inner hexagon is centered within the outer one with its orientation at right angles to the orientation of the outer one. Its size is chosen so as to make the outer hexagon three times the area of the inner hexagon (so there is a linear scaling factor of 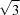). There are, up to rotational symmetry, only two double hexagons (of any particular size) (see Fig. 4). When three double hexagons meet at a vertex, the gray parts around the vertex form another hexagon. We call these types of hexagons *corner hexagons*.

Suppose that we have a hexagonal tiling of the plane with double hexagons. By this we mean that we are using the outer hexagons as the tiles. Suppose that from the perspective of the arrowing on the outer hexagons this hexagonal tiling is well matched. Three outer hexagons meet at every common vertex *v*, and the three edges of the corresponding inner hexagons that are closest to *v* form three edges of a corner hexagon *H* centered on *v*. With the terminology introduced above we can ask whether or not these three arrows are mixed. If they are mixed then we can extend the arrowing to make *H* well arrowed.

**Figure 2:**
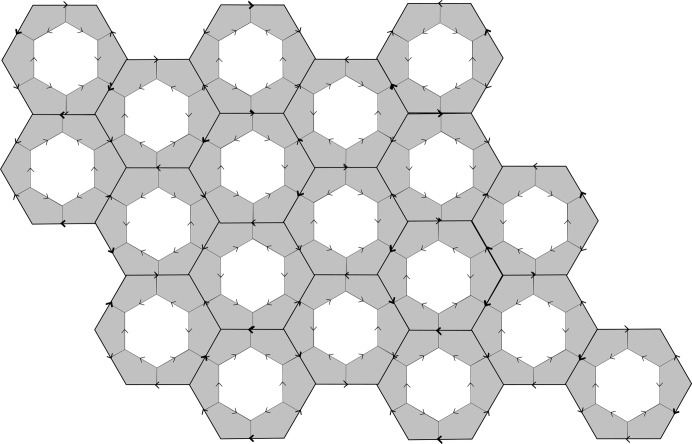
A legal patch of double hexagon tiles. Where three double hexagons meet at a vertex, a corner hexagon is created. Note the mixed arrowing of these small gray corner hexagons.

Suppose that all the corner hexagons of the tiling can be well arrowed in this way. Collectively, the inner hexagons together with the corner hexagons form another hexagonal tiling of the plane, if we ignore the question of their arrows matching. However, there does arise the question of whether or not all the common edges of adjacent small hexagons actually do have matching arrows, that is, whether or not this new tiling is well matched. The double hexagon tiling is called *legal* if they do. Thus, a double hexagon tiling is legal if its outer hexagons are well matched, all of its corner hexagons can be completed to well arrowed hexagons, and the consequent well arrowing of the small hexagons completes to a small hexagon tiling of the plane which is well matched. In this situation we have two well matched hexagonal tilings, one using the large hexagons and the other using the small ones, inner and corner hexagons. Fig. 2 ▸ shows a patch of a legal double hexagon tiling.

The double hexagon tiles that we are discussing are also called *Penrose hexagonal tiles* and a legal tiling is a *Penrose hexagonal tiling*. We shall use both names in this paper, because within the context of understanding the intimate relationship between Penrose hexagonal tilings (based on the large hexagons) and Taylor–Socolar hexagonal tilings (based on the small hexagons), it is convenient at times to simply think in terms of legal double hexagon tilings.

## 3. Decorations and triangles   

**Figure 3:**
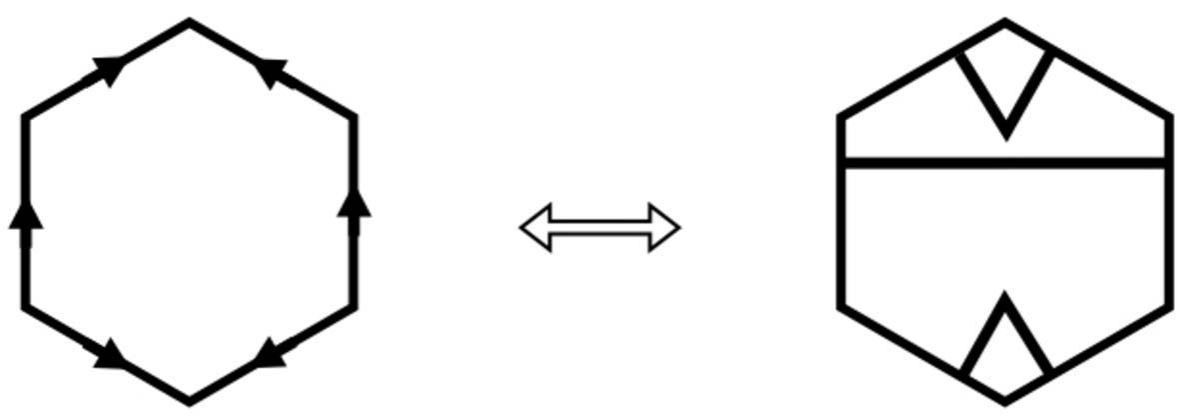
Well arrowed hexagonal tiles can be converted into hexagonal tiles with stripes. These decorations fit together to make triangulations of the plane.

There are other decorations of well arrowed hexagons and double hexagon tiles that are equivalent representations of the arrowing but help to make the underlying geometry of the tilings more transparent. The first of these is the marking of well arrowed hexagons shown in Fig. 3 ▸, which replaces the arrows of a well arrowed hexagon with a black stripe and two black corner markings (see Socolar & Taylor, 2011 ▸). Initially, we will use this representation of the arrowing with the small hexagons, and later for the outer hexagons.

Notice that when two well arrowed hexagons are attached along some edge so that the corresponding arrows match (*i.e.* they are well matched), the black markings line up, either stripe to stripe, stripe to corner, or corner to corner, to create an extended black path. In fact, that the stripes and corner markings match to form extended paths is exactly the same as arrow matching. In the resulting paths the corner markings indeed serve as corners at which the direction of the path changes. If we have a well matched tiling of well arrowed tiles, we will have also a set of paths. It is easy to see that if, in following a path (see Fig. 18 as an example), it turns right or left at a corner, then at its next corner it will turn in the same sense (again right or again left) and so the resulting paths will be equilateral triangles (the corners create 60° angles).

The only way this can fail is if there are paths that extend infinitely in some direction along some straight line. A tiling with such a path is called a *singular* (or *non-generic*) tiling. Later on we will examine the similar paths created by the stripes and corner markings on the large hexagon tiles, and the same issue of singularity will arise.

The generic situation is that of *non-singular* (or *generic*) tilings, that is, all of the paths form triangles. In this paper, in order to keep all the essential ideas clear, we shall always assume non-singularity, though at this point we need it only with the small hexagons and their markings. The resulting triangles come in various sizes and arrangements, and this is something we address in the next section.

**Figure 4:**
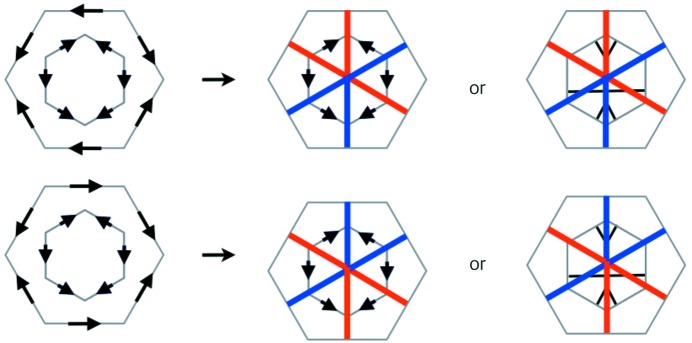
Arrows on the edges of the outer hexagon which are oriented in the counter-clockwise direction are represented by short red half-diameters. For arrows in the clockwise orientation we use short blue half-diameters. If we include the striping decoration of the inner hexagons as well (Fig. 3 ▸), we arrive at the fully decorated double hexagons shown on the right-hand side of the figure. Evidently the decorated tiles carry information fully equivalent to the arrowing. Notice that proper matching of the edges of outer hexagons is equivalent to a *change* of color as the short diagonal passes through the common edge. Also notice that if one holds the black stripe horizontally, then as one moves along a full blue diagonal from right to left, the diagonal passes through the black stripe from above to below. It is the other way around for red stripes. We use this observation to make the color determination of the short diagonals in Fig. 10 ▸.

The second decoration is one that we make to double hexagon tiles, replacing the outer arrowing by colored short diagonals (short diagonals are the ones that pass at right angles between opposite edges, as opposed to long diagonals that pass from vertex to opposite vertex). This is explained in Fig. 4 ▸.

If we begin with a legal double hexagon tiling then we know that we end up with two well matched hexagonal tilings: one of large hexagons and one of small hexagons. Since we are assuming non-singularity, the well matching of the small hexagons leads to a collection of triangles on the plane – equilateral triangles created by the stripes on the small hexagons. Each small hexagon has an inner part of some edge of a triangle across it and the corners of two other triangles, one on each side of that edge, so altogether each small hexagon is involved with three triangles.

The very smallest triangles (called level 0 triangles) are those composed by putting three corner markings together around a common vertex of three small hexagons. Every stripe in a hexagon obviously belongs to a triangle larger than these smallest ones. Indeed there are triangles of ever-increasing sizes, without limit. It is this result that we will establish in the next section.

## 4. Nesting and hierarchy   

Let us continue with a non-singular legal double hexagon tiling 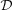, in which we have completed its small hexagons to a well matched hexagonal tiling and then resolved everything into triangles by decorating each of the small hexagons.

**Figure 5:**
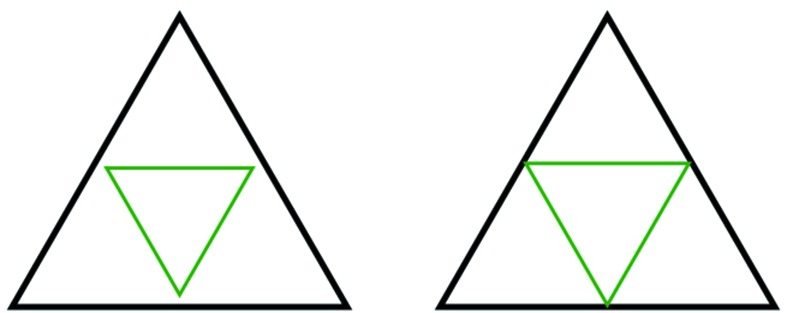
The green (smaller) triangle is nested in the larger (black) one. Notice the two ways of drawing this. A patch of triangles that arise from our tiling presents the inner triangles as being totally in the interior of the outer ones, as shown on the left-hand side here. We often will wish to allow the inner triangles to stretch to meet the boundaries of the outer triangles, as shown on the right-hand side of the figure. This creates three new triangles called *corner triangles*, so that the outer triangle is now decomposed as four equal-sized triangles.

A triangle is *nested* in another one if it appears as in Fig. 5 ▸, where the smaller triangle is nested in the larger. In this section we will prove that except for the very smallest triangles (the ones made from three corners) every triangle has another one nested inside it. From this, we will see that every triangle has inside it a sequence of triangles nested within each other, diminishing in size to the smallest-size triangles. We refer to this phenomenon by saying that all triangles are *nested within*.

There is more to this. Let us stretch out, or expand, the triangles created by the decorations of the small hexagons (inner hexagons and corner hexagons) so that the corners meet the edges of the triangle surrounding them, as illustrated in Fig. 5 ▸. In doing this each nested triangle produces three neighbors that fill out the whole triangle that it lies in. In fact, there is a nesting that involves one triangle sitting inside another of exactly twice the linear size, so that the larger triangle is decomposed into four equal-sized equilateral triangles of which the nested triangle is one. The three triangles that emerge as neighbors of the nested one are called *corner triangles* (not to be confused with the smallest triangles that we formed out of three corner markings).

**Figure 6:**
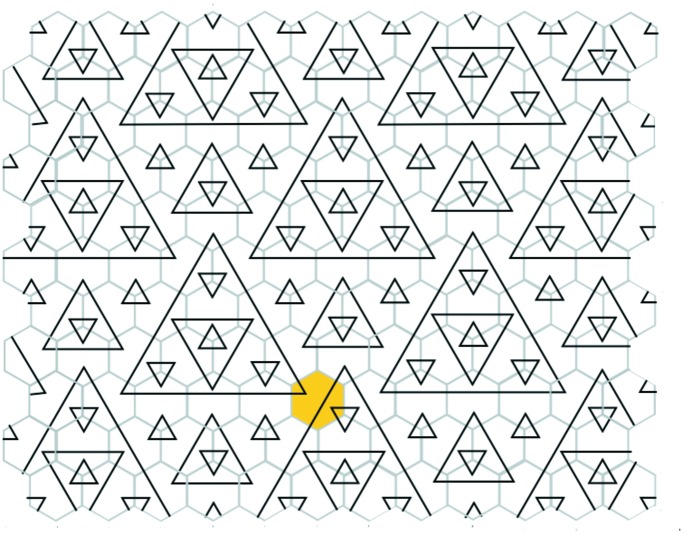
Periodic arrangements of triangles are possible if only decorations of the small hexagons are used.

We will speak of the patterns of triangles (expanded or not) which are formed by the decorations of the small hexagons as *arrangements of triangles* and derive their nesting properties as we proceed. Notice that without the implications derived from the decorations of the outer hexagons it is possible to get an arrangement of triangles like the one shown in Fig. 6 ▸, which is visibly periodic.

In all, we shall see that triangles that are nested within appear on ever-increasing scales, so there is a hierarchical structure. We shall call such an arrangement of triangles a *nested triangulation*.

When we refer to the sizes of triangles in one of the arrangements of triangles, we will always refer to the side lengths of the stretched-out versions. Lengths are normalized so that the smallest triangles will be of side length equal to 1. We will see that with this normalization all lengths are powers of 2.

In the sequel we will commonly use both versions of the triangles and nested triangles that emerge from our discussion – the original ones that come from the decorated hexagons and the stretched-out ones that give us the arrangements of triangles. Once we have proved that all triangles are nested within, it is trivial to convert from one picture to the other.

**Figure 7:**
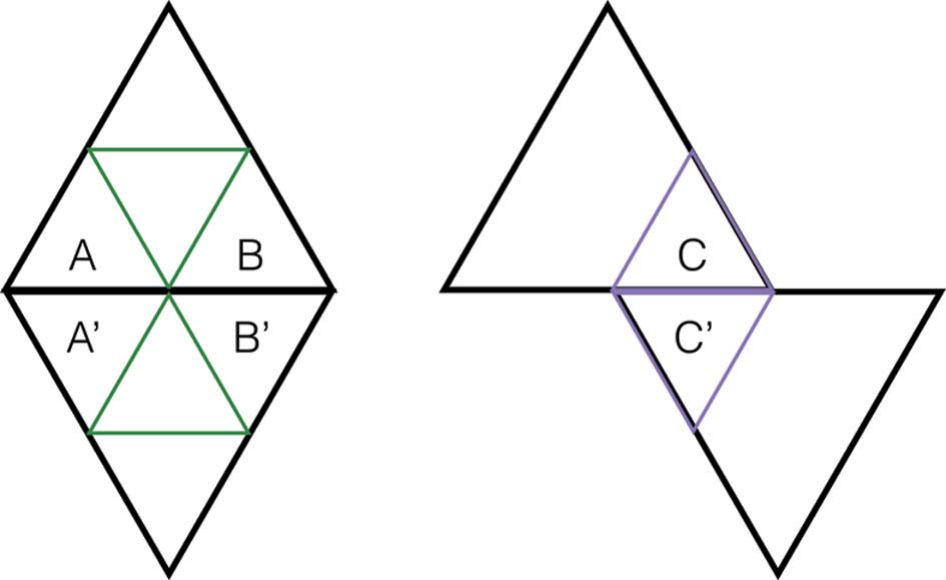
On the left side the black triangles form an opposite pair of triangles. So too do the pairs of smaller triangles labeled 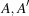 and 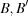. On the right-hand side the two black triangles do not form an opposite pair, but the two purple triangles 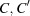 do.

In the stretched-out version, two triangles are said to make an *opposite pair* if they are of the same size and share a common edge (see Fig. 7 ▸). Notice that there is no specification of how each of the two opposing triangles fits into the overall arrangement of triangles.

**Proposition 4.1**

Let 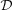 be a non-singular legal double hexagon tiling. Complete its small hexagons to a well matched hexagonal tiling and let 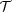 be the resulting arrangement of expanded triangles formed from the decorations of the small hexagons. Then

(i) all triangles occur in opposite pairs;

(ii) the side lengths of the triangles are all of the form 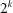 for some 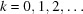 (*k* is called the *level* of the triangle);

(iii) every triangle is nested within.

The proof of proposition 4.1[Statement proposition4.1] is by induction on the size of triangles. The smallest triangles have side length 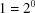 (level 0). There is no nesting within to take place. The stretched triangle pattern created by these triangles is in itself a genuine triangular lattice of the plane and, in particular, every triangle edge borders an opposite pair of triangles.

We now assume that the three statements of proposition 4.1[Statement proposition4.1] have been proved up to some level *k*.

**Figure 8:**
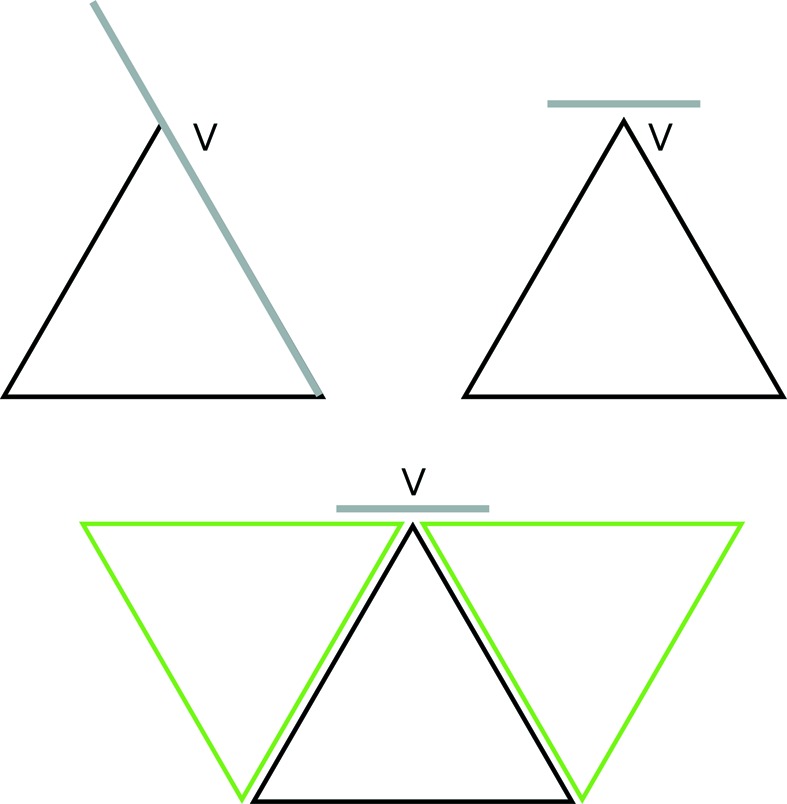
*v* is a vertex of a triangle (shown in black) of level *k*. At *v* there is a small hexagon and its stripe allows for only two things to happen: either one of the edges of the triangle extends through *v*, in which case it is an edge from a larger triangle, or there is an edge passing through *v* that is parallel to the opposite edge of the triangle. In the latter case we use (i) of proposition 4.1[Statement proposition4.1] to place down the two opposite pairs of triangles of adjoining triangles, shown in green (the adjacent edges are actually coincident edges of course). Then we see that the edge through *v* must actually be an edge that includes both the top edges of the green triangles: thus again a larger triangle.

First we check that there must be triangles of size larger than 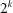. Fig. 8 ▸ shows why. It shows a triangle of level 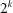 and uses matching triangles to see that there must be larger ones.

**Figure 9:**
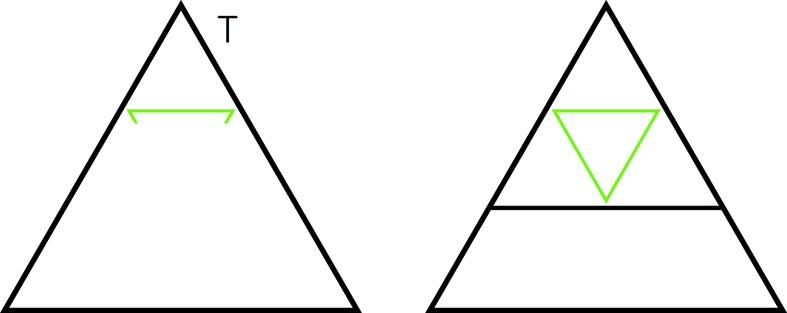
We are given a triangle *T* of minimum size larger than 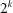. From one of its vertices (we have taken it as the top one here) we fit in the largest sub-triangle possible (at the very least, there is always a triangle of level 0 that can be fitted in). Its lower side is indicated in green. It must turn inwards at the sides of *T* and complete to the opposite green triangle. By the induction assumption its other two sides also complete to opposite pairs, and this leads to the new black triangle with the green triangle nested in it. Since we started from a maximal-sized sub-triangle, this larger black triangle must in fact be the entirety of our original triangle *T*. This shows that *T* has edge length 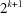. The visible nesting and the induction hypotheses show that the new triangle is nested within.

We now take any triangle *T* of the next size, say *m*, that is larger than 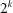. We see immediately that 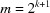 and it is internally nested, in the right way (Fig. 9 ▸). This completes the induction steps for parts (ii) and (iii).

**Figure 10:**
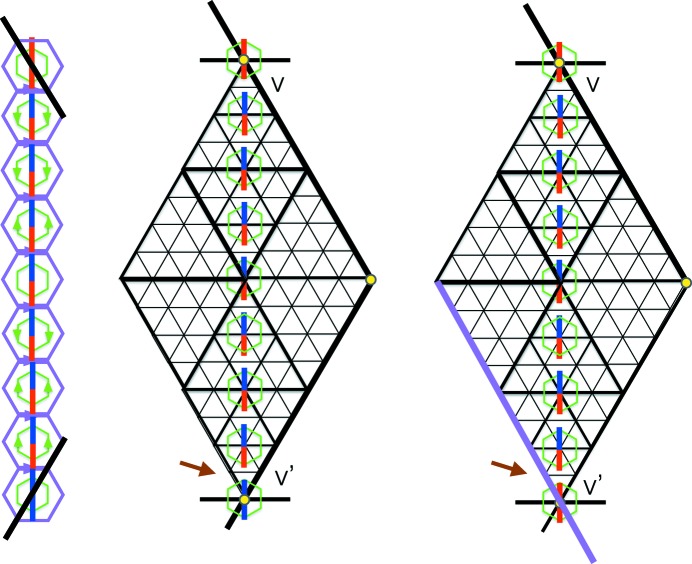
In the center of the figure we see the large pair of triangles making a diamond shape between the two extreme vertices *v* and 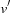, which are assumed to be vertices arising from centers of the large hexagons of the double hexagon tiling. The diamond is made up of an opposite pair of fully internally nested triangles. The triangles are all in their stretched form, but the line thicknesses indicate the nesting relationships. On the left side we see the corresponding arrangement of double hexagons that surround the small hexagons between *v* and 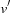. The arrows must match, but their common orientation in the horizontal direction is irrelevant here. There is a color change as we cross each triangle edge at right angles. The key point is what happens at the ends of the diamond as the color line crosses edges (indicated by the thickest lines) which are not at right angles to it. The main edge at *v* is shown by the heavy black line. The rules for coloring hexagons show that the color stripe is fully red here, see Fig. 4 ▸. The main edge at *v* has to be matched with its partner at 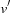, where the color strip changes to fully blue. Notice the correct color change at the arrow. The scenario shown on the right side of the figure, where the main edge at 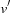 is shown in purple, cannot occur because of the color change violation at the brown arrow.

We now come to the proof of part (i). It is useful to prepare this by looking at the situation pictured in Fig. 10 ▸. What this shows is how the coloring of the tile decorations is related to the matching of opposite triangles. The color rules show that as a color diagonal crosses a triangle edge at right angles it changes color. When it crosses an edge that is not at right angles to it then it does not change color, but, as we have noted in the caption to Fig. 4 ▸, its color is related to the way in which it crosses the edge. This figure is the basis for our proof of matching triangles.

**Figure 11:**
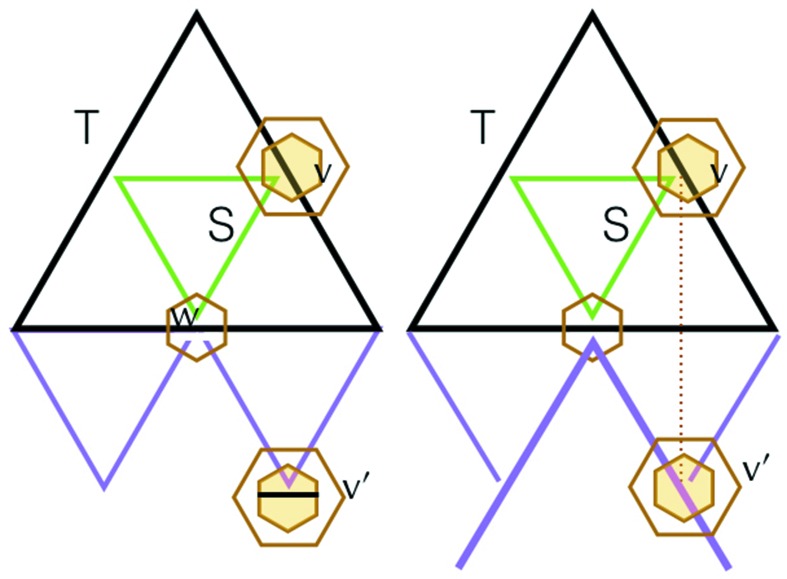
The vertex *v* is the unique vertex of triangle *S* which is the center of a double hexagon tile. The two lower corner triangles formed in *T*, with common vertex *w*, have opposite triangles shown in purple. The question is, what happens at the vertex 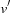
? Neither of the two possibilities shown here can occur. On the left, the triangle with vertices 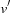 and *w* would be left unclosed at *w*. On the right we are in the situation shown on the right side of Fig. 10 ▸, which we know violates the color change property.

Continuing to the proof of (i), we start with a triangle *T* of level 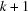 and show that it must be matched by a triangle of the same level on each of its sides. Let *S* be the largest equilateral triangle nested in it. Now, any equilateral triangle of any level 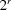 in our arrangement of triangles has exactly one vertex at the center of a large hexagon of the double hexagon tiling. This has to do with the lattice structure induced by the arrangement of triangles coming from the double hexagon tiling, and though pretty self-evident in the figures, is explained algebraically in §5. In Fig. 11 ▸ we have made such a choice, indicating it by the small yellow hexagon at *v*. We shall use this coloring convention to mark other vertices that are centers of the large hexagons. We shall start by showing that there must be matching triangles to *T* on the two sides of *T* on which *v* does not lie.

**Figure 12:**
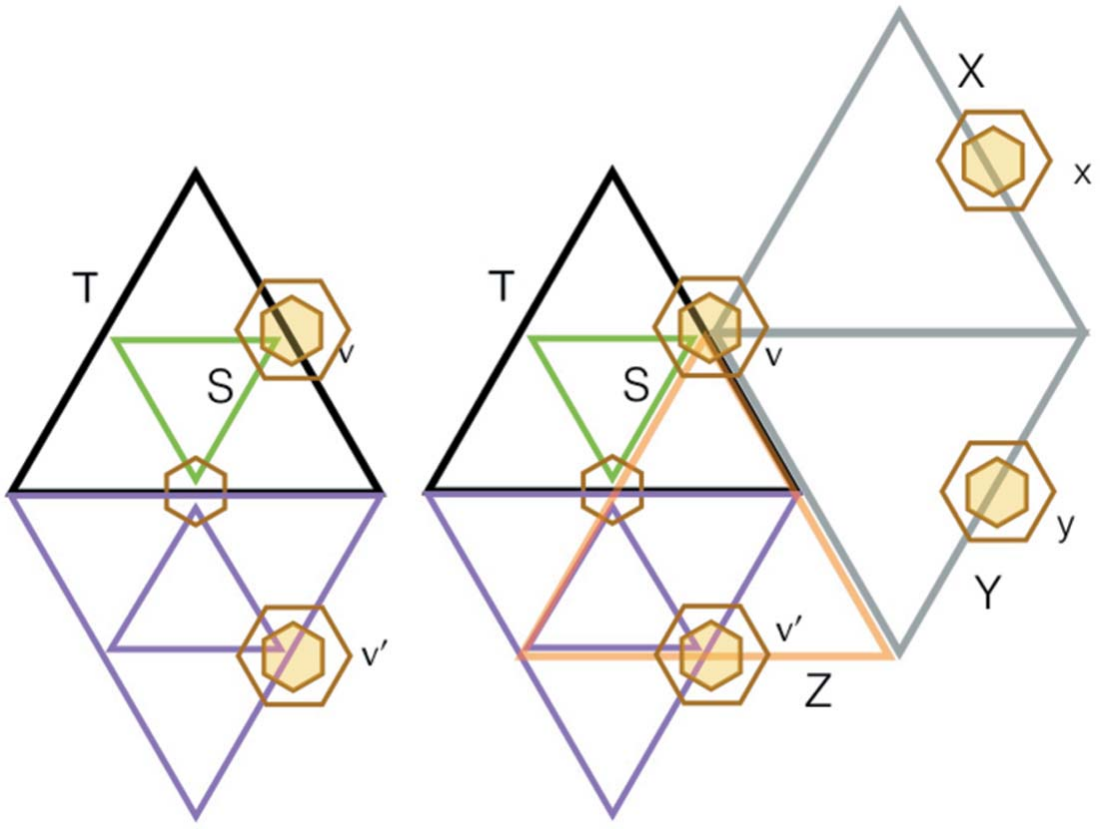
The left-hand side shows the matching that has to take place on the lower side of *T*. The right-hand side shows what happens if there is not an opposite match to *T* on the side containing *v*. This corresponds to the right-hand side of Fig. 11 ▸. Chasing around the pairs of opposite matching triangles yields 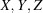 and the latter is clearly totally mismatched with *T*.

The triangle *S* creates a partition of *T* into itself and three surrounding triangles, and we know that each of these must have an opposite match. We show these matching triangles along the lower edge of the *T* solid edge. The shape of the small hexagon at their intersection *w* must be of the type shown in Fig. 11 ▸. What does the small hexagon at 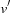 look like? The caption to Fig. 11 ▸ shows that neither of the two possibilities shown there is possible. Thus, the remaining possibility, which is that of a matched triangle for *T*, must occur. This is illustrated on the left side of Fig. 12 ▸. This same argument can be applied to the other side of *T* which does not contain the point *v*.

There remains the task of proving that the side that contains *v* also matches *T* to a triangle of the same size. Let us suppose that on this side the matching fails. The argument we have just used tells us that in this case on this side we will see a triangle *X* which aligns its corner at the point *v*. The point *x* is the center of a double hexagon, just like *v* was, so it follows by what we have proved that the triangle *Y* shown exists and matches it. Again, it has a point *y* which is a double hexagon center and so *Y* produces the matching triangle *Z*. But this is clearly a contradiction since *Z* overlaps but does not coincide with the triangle *T*. This contradiction shows that there is a matching opposing triangle along the edge of *T* containing *v*.

With this we conclude part (i) of proposition 4.1[Statement proposition4.1] and so the entire proposition.

□

Since there are triangles of every level, it is impossible that there are any translational symmetries.

**Proposition 4.2**

Every non-singular legal double hexagon tiling is aperiodic.

**Figure 13:**
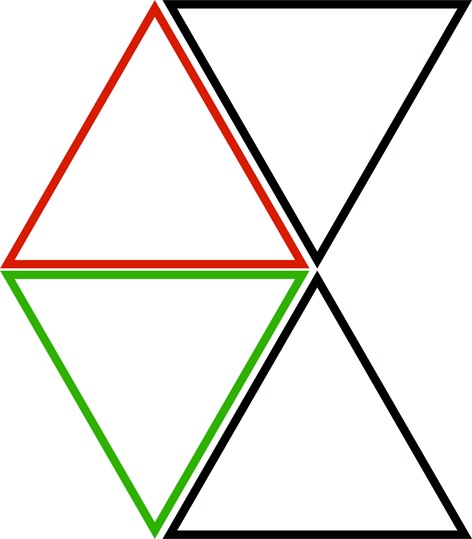
The two triangles whose corners make up the pair of corner markings on a well arrowed tile are always of the same size. Here the pair of triangles is shown in black. The matching on opposite sides of triangles leads to the red and green matched triangles. Since these too must match, we see that all four triangles are of the same size. Note that there is no presumption here about how these triangles lie in larger triangles.

Looking at Fig. 13 ▸ we see:

**Proposition 4.3**

In any small hexagonal tile the triangles that arise from its two opposite corner markings are of the same size.

For future reference we also note (see Fig. 5 ▸):

**Proposition 4.4**

In the nested triangulation created by the standard edge and corner markings of arrowed hexagonal tiles, every stripe forms the central part of an edge of some triangle.

## 5. The algebra of nested triangulations   

**Figure 14:**
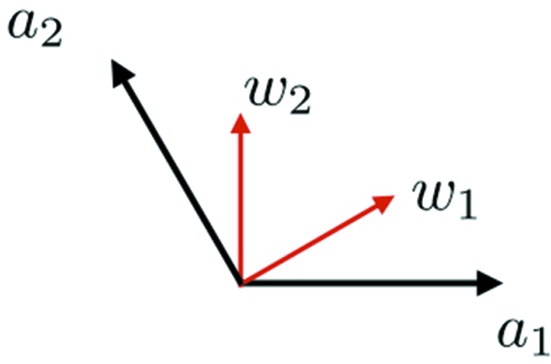
The basis vectors for the lattices *Q* and *P*. The directions of edges in the *Q* triangulations are 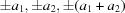, which are called *a* directions, and those of *P* triangulations are 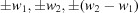, which are called *w* directions.

**Figure 15:**
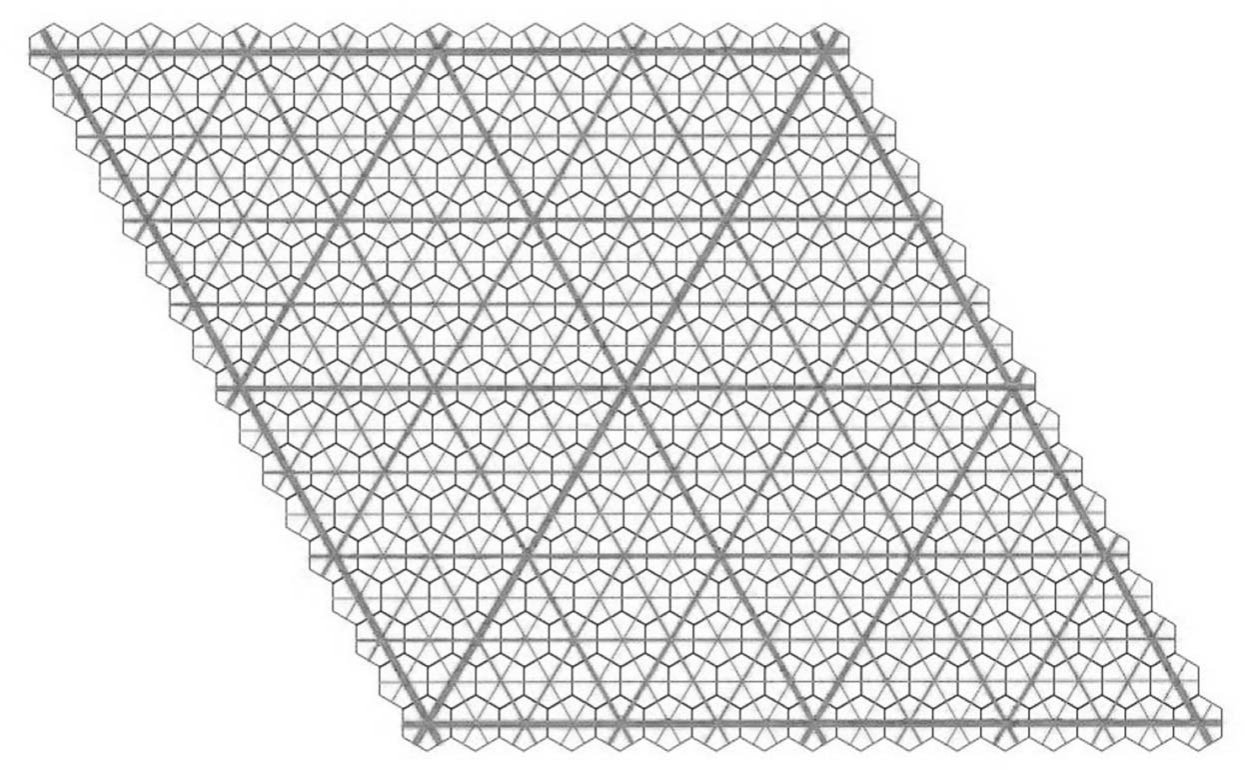
A nested triangulation of the plane. Triangulations of increasing scales (four are shown here) coexist in one underlying triangulation. Each increase in scale can be created in four different ways by choosing one vertex of the previous scale as a reference point.

If we start with a non-singular legal double hexagon tiling then we obtain a tiling of the plane with the small hexagons. The centers of these hexagons form a triangular lattice of the plane composed of level 0 (side length 1) equilateral triangles, as we have seen. For definiteness we now specify this lattice as a set of points in 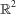, namely the set of points 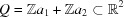, where 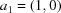, 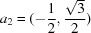 (Fig. 14 ▸). Joining nearest neighbors of *Q* produces the triangular lattice of level 0 triangles, indicated by the thin lines in Fig. 15 ▸.

We know that there are also triangles of level 1 (side length 2). They are matched across each of their edges, and so there is a second triangular lattice of the plane by equilateral triangles. This meshes precisely with the first, in the sense that each level 1 triangle is composed of four level 0 triangles. The vertices of the level 1 triangles form a coset 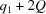 of *Q*.

We may repeat this process, now looking at triangles of level 2, whose vertices lie on a coset 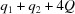 (where 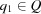 and 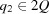). Continuing this way we are led to view our nested triangulations in terms of ever-refined cosets from the sequence
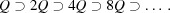
Thus, the double hexagon tiling leads to the sequence
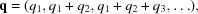
where each 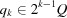. We refer to such a sequence as a *Q*-*nested triangulation* 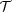. Indeed, we see that any such sequence corresponds to a sequence of triangular lattices with each level nested within the next, that is to say every triangle of one level appears as a corner triangle or as a central triangle within a triangle of one level higher. Specifically, up to rotation a typical triangle of level *k* has vertices 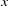, 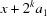, 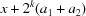, all of them lying in one coset 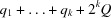, where we assume 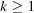. The midpoints of its edges are 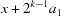, 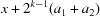, 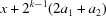, which form the vertices of a triangle of level 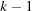 in the coset 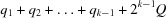, hence the nesting.

The sequence 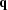 can be interpreted as a *Q*-adic element of the inverse sequence
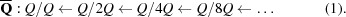
This sets up a bijective correspondence between *Q*-adic numbers and nested triangulations, and we will write 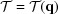 when we wish to make the connection explicit. In the sequel 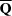 will be written as 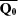, the first in a series of such inverse limits.

The condition of non-singularity has algebraic consequences. The *Q*-nested triangulation is singular if some of the paths created by the stripes of the small hexagons do not close, but rather extend indefinitely. Since the directions of the stripes are all in the *a* directions of the lattice *Q* (see Fig. 14 ▸), this is equivalent to saying that there is an infinite path of consecutive edges in some direction *a* 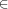 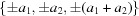 and this in turn implies that 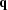 lies in 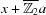 for some 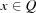. Here 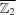 is the 2-adic integers. We need to avoid 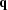 having this form. See Lee & Moody (2013 ▸) for more details.

In order to interpret the double hexagon tiles in this algebraic setting, we need, along with *Q*, its 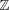-dual *P*, relative to the standard dot product on 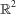. Thus 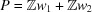 where 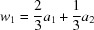 and 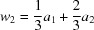 (Fig. 14 ▸). We note that
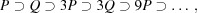
all the steps being of index 3. In fact, each of the lattices in this chain is a scaled and rotated version of the one before it, and in particular a scaled and rotated version of the original triangular lattice *Q*. They are all triangular lattices. We do not use *P* directly in what follows, but rather 3*P*, since we wish to keep everything inside the initial lattice *Q*.

There are two clear differences between the triangular lattices arising from *Q* and 3*P*. The first is that the basic triangles in 3*P* have side length 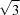, so factors of 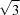 relate scales of *Q*- and 3*P*-nested triangulations. The second is that the directions of the sides in all the *Q*-nested triangulations (at all scales) are 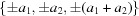, which we are referring to as *a* directions, and those for 3*P*-nested triangulations are 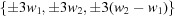, which we call *w* directions. These two sets of directions are interchanged by 90° rotations.

In a legal double hexagon tiling the large hexagons enclose one third of the vertices of the small hexagons, and their centers form some coset 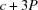 of 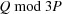. This brings us to a second inverse sequence of groups based on 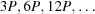 and corresponding group 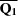 which is related to 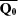 as shown in the commutative diagram (2). All the mappings here are the natural homomorphisms that arise from factoring out larger subgroups.
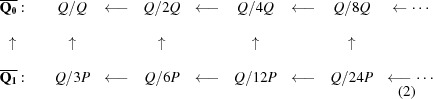
Given the choice of 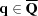 and 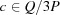, there is a unique element
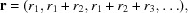
where 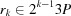 for each *k*, which maps onto 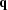 and has 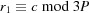. This follows from the more general fact:

**Lemma 5.1**

For all 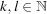,
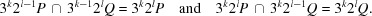

Proof: dividing out common powers of 2 and 3, we are reduced to proving that 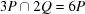 and 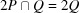, respectively, both of which are trivial to verify.

□

So, given a non-singular legal double hexagon tiling, we arrive not only at an element 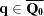 but also an element 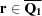. This element picks out one coset 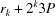 for each 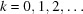 and so should determine a family 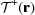 of nested triangulations, just the same way as 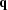 did. To guarantee that we really do have a non-singular 3*P*-nested triangulation, that is to avoid infinite lines, we have to make an assumption similar to the non-singularity assumption that we have already seen. This time the triangle edges are in *w* directions, so we must assume that 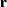 does not lie in 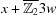 for any 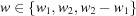.

Thus, the joint conditions equivalent to non-singularity are that for all 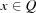:

(i) 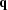 does not lie in 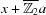 for any *a* 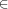 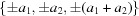;

(ii) 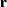 does not lie in 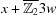 for any 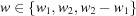.

These are the same conditions as appeared in Lee & Moody (2013 ▸), though we did not use double hexagon tilings there. We will pursue the detailed study of the singular double hexagon tilings in another paper.

Returning to our discussion, the situation is this. We are given a generic legal double hexagon tiling whose small hexagons are centered on *Q* and whose large hexagons are centered on 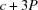. We thus have two nested triangulations 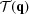 and 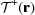, the first being determined by the markings on the small hexagon tiles and the second simply by the algebra of the commutative diagram (2). Since the large hexagon tiles can be given their own stripe and corner markings based on their arrowing in just the same way as we did for the smaller hexagons, it is natural to ask whether or not this new nested triangulation based on 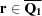 is the one that these markings create. In fact, this is indeed the case. Here is the argument. As a matter of nomenclature, we will use the same concept of *level* for the new triangulation 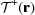 as we did before. The smallest triangles are said to be of level 0 with side lengths equal to 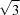, and subsequent sizes have levels 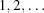 of side lengths 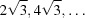.

**Figure 16:**
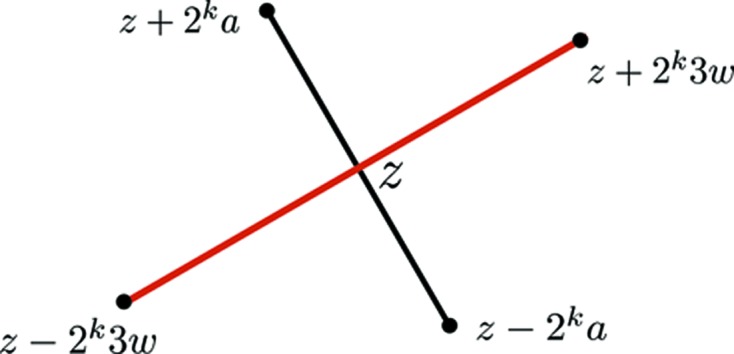
How triangle sides of triangles from 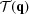 and 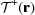 right-bisect each other.

Take any edge *e* of a level 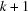 triangle 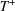 from the triangulation 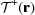 and let *z* be its midpoint. The two ends of *e* have the form 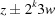 for *w* in one of the *w* directions of the lattice (see the red line segment in Fig. 16 ▸). Since both end points lie in 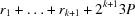, we see that *z* 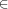 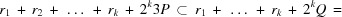 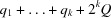 and 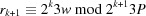. Let *a* be in the *a* direction at right angles to *w* and consider the two points 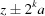. These are two points of a level 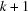 triangle of 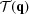 and *z* is its midpoint.

To see this explicitly we take the case where 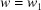 and 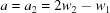. Then
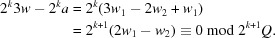
This shows that
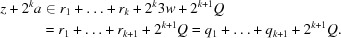
See Fig. 16 ▸. This proves:

**Proposition 5.2**

At their midpoints, the edges of level 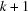 triangles of 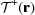 both right-bisect and are right-bisected by edges of level 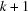 of 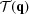.

**Figure 17:**
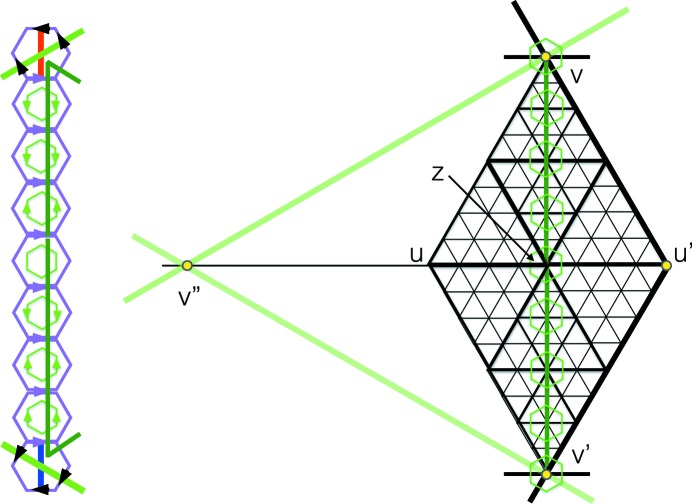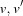 are the ends of an edge *e* of a triangle 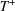 from the nesting determined by 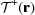. At its midpoint *z* we see the edge 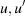 of a triangle *T* from 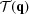. The black triangles all come from the triangulation of 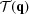, the largest ones being of level 3. The edge *e* is maximal, in the sense that it is not part of an edge of some larger triangle from 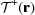. Thus, at its ends, the stripes of the large hexagons at *v* and 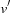 are in the directions of the other sides of 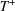. The inner hexagons along 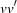 are centered at double hexagon centers and their stripes are all oriented in the same direction, namely perpendicular to 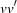. At the left we have separated out the outer hexagons that overlay the small hexagons along 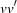. We see their matching arrows and how their stripes align to form the edge 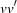 (in green). The colors (red/blue) of the short diameters of these large hexagons are determined by (or determine, whichever way one wants to put it) the color rule that we see in Fig. 10 ▸, though note that the stripe of the large hexagons is perpendicular to that of the small ones, so the right/left crossing rule is opposite! The fact that the stripe orientation changes at the end dictates that the edge 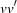 is an interior edge of a larger triangle. The shift indicated by the orientation of the arrows matches this.

These midpoints are centers of double hexagon tiles and, as in all double hexagon tiles, the stripes of the inner and outer hexagons are at right angles. This applies to triangles of all levels 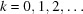. Since by proposition 4.4[Statement proposition4.4] every stripe of a small hexagon lies at the middle of some edge of some triangle, we conclude that the nested triangulation 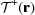 is directly related to the stripes on the large hexagon tiles, namely the path created by these stripes forms the triangles of this triangulation. Thus the triangular tiling produced by the outer hexagons is indeed the one produced by the nested triangulation of 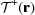. Fig. 17 ▸ illustrates what is going on here and also shows that the edge shifting (involved in truly nesting the triangles) is properly indicated by the outer hexagon tiles.

A direct consequence of proposition 5.2[Statement proposition5.2] is that generic (respectively, singular) 3*P*-nested triangulations give rise to generic (respectively, singular) *Q*-nested triangulations:

**Proposition 5.3**

Let 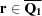 and let 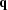 be its image in 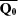. Then 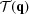 is generic if and only if 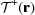 is generic.

## 6. From nested triangulations to double hexagon tilings   

**Figure 18:**
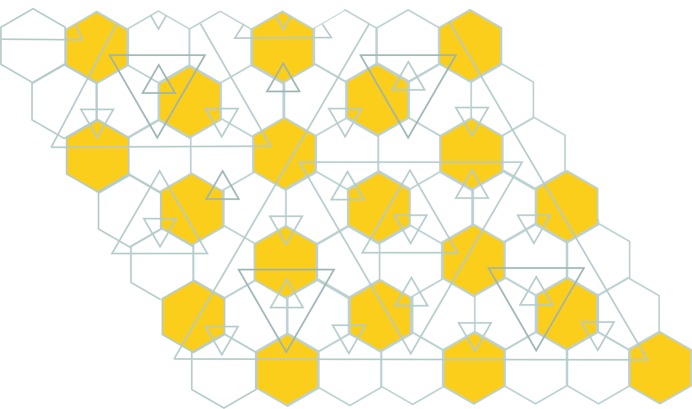
Starting from the pair 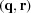, with 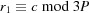, hexagons centered at the lattice points of *Q* are shown, with those centered on a coset 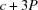 indicated in yellow. The nesting of the triangulation 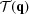 arising from 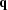 is indicated.

At this point it is rather clear that given any triangulation 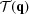 and any choice of one coset 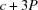 leading to 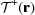 it ought to lead to a legal double hexagon tiling. Here are the details. We will assume that both triangulations are non-singular. This guarantees that there are no infinite edges, we get proper triangulations, and they are nested. This nesting can be geometrically manifested by laterally shifting the edges as indicated by the nesting. The Voronoi cells of the lattices (nearest neighbor cells) are hexagons centered, respectively, on the points of 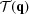 and 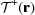. We know that every hexagon from 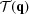 has a triangle edge passing through it and this edge will be shifted laterally in nesting. This is shown in Fig. 18 ▸. The hexagon is made into a well arrowed hexagon by placing the pair of parallel arrows in the direction of the shift. The small hexagons now make a well arrowed and well matched hexagon tiling.

**Figure 19:**
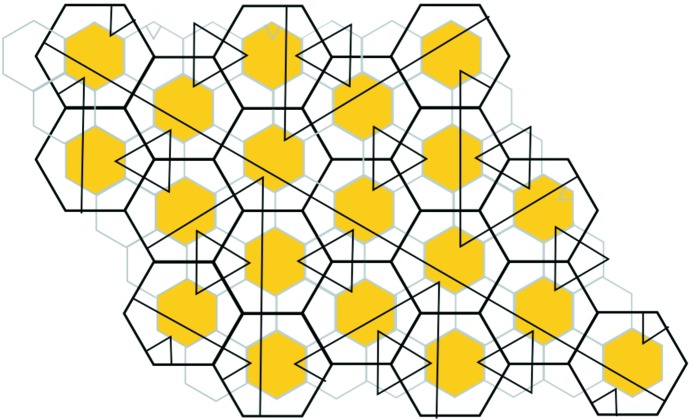
Continuing from Fig. 18 ▸, from 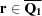 we obtain a nested triangulation 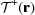, part of which is shown here.

Now we do the same thing with the triangulation 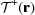, leading to the new hexagonal tiling, again with arrows indicating edge shifting in the nesting (see Fig. 19 ▸). This is the second well arrowed and well matched hexagonal tiling. The outcome is that we have a tiling of double hexagon tiles which is non-singular and legal (see Fig. 2 ▸).

## 7. Penrose tilings, Taylor–Socolar tilings, and beyond   

**Figure 20:**
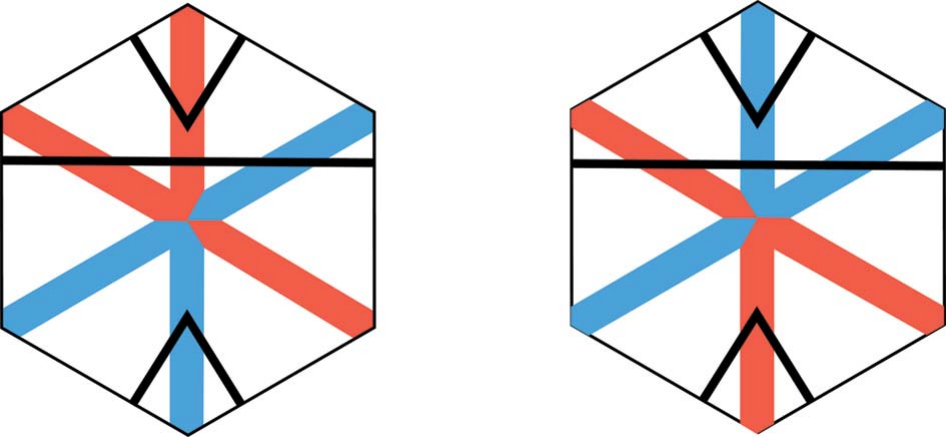
The two prototiles for the Taylor–Socolar tilings.

By definition, a Penrose tiling is precisely a legal double hexagon tiling. Taylor–Socolar tilings (T–S tilings) are usually defined by the T–S tiles shown in Fig. 20 ▸ and they are assembled as regular hexagonal tilings, but under the matching rules:

*RT1* the black lines must join continuously when tiles abut;

*RT2* the ends of the diameters of two hexagonal tiles that are separated by an edge of another tile must be of opposite colors.

**Figure 21:**
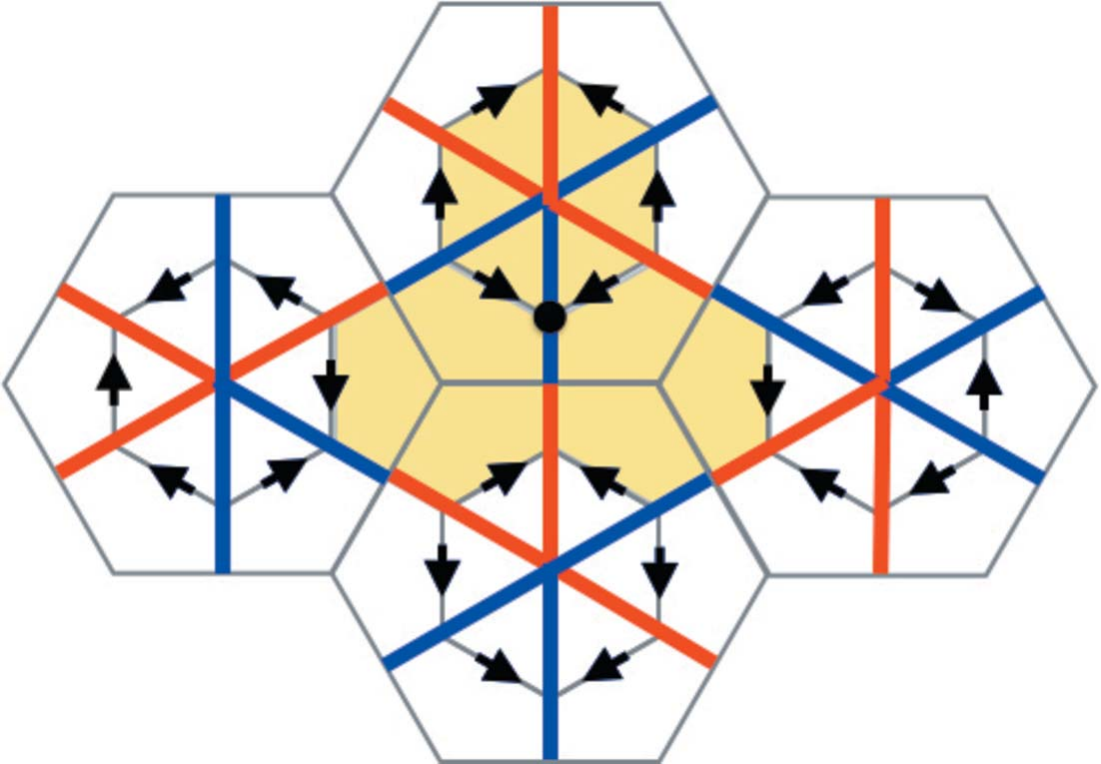
A small patch of double hexagon tiles with the new decoration shown on the right of Fig. 4 ▸.

In Fig. 10 ▸ we have seen that the diagonals of the inner hexagons of a double hexagon tiling can be colored, and if we restrict this coloring to the actual physical area of the inner hexagons then we have the colorings of Fig. 20 ▸. The matching of the outer arrows of the double hexagon tiling amounts to the color rule *RT2*, so we have in this way one third of a T–S tiling (Fig. 21 ▸). If the double hexagon tiling is legal, then we know that this partial hexagonal tiling of inner hexagons along with the corner hexagons completes to a new properly arrowed hexagonal tiling together with a corresponding nested triangulation. If we assume that the nested triangulation is non-singular, which is generically the case, then this tiling-triangulation corresponds to a unique T–S tiling. That is, the one-third tiling we have completes uniquely to a T–S tiling. The proof of this is given in Lee & Moody (2013 ▸) – each non-singular nested triangulation corresponds to a unique T–S tiling and *vice versa*.

Thus, every non-singular Penrose tiling produces inside it a non-singular T–S tiling made out of its inner and corner hexagons. Now let us go in the other direction. If we begin with a non-singular T–S tiling then it produces a nested triangulation out of the stripes on each hexagon. Relative to a fixed coordinate system, this triangulation corresponds to an element 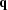 in the *Q*-adic completion 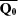. In order to obtain a double hexagon tiling from this, we need to select which hexagons will be the inner hexagons and which the corner hexagons for the new tiling. This amounts to choosing one coset from the 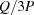. Choose one, say, 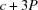. Then there is a unique 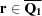 that maps 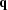 under the natural mapping of 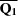 to 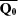 and for which 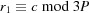. This produces the centers and the nested triangulation that determines a legal double hexagon tiling, as we have pointed out in §6.

To reiterate, we see that the nested triangulation of the large hexagon tiles determines the nested triangulation of the inner hexagonal tiles. Thus, although we only see the coloring of one third of an underlying T–S tiling, the entire nesting of the triangulation arising from the small hexagon tiles is implicitly known from the nested triangulation of the larger hexagon tiles: we know 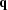 once we know 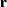.

A noteworthy observation comes by comparing Fig. 20 ▸ and Fig. 4 ▸: it shows that the distinction between the parity [that is, the difference between the two types of small hexagon tiles (respectively, large hexagon tiles)] is the same for the T–S tiling and the Penrose tiling. Thus the parity distribution of a Penrose tiling is the same as the parity distribution of one coset modulo 3*P* of the T–S tiles.

Although it is shown in Baake *et al.* (2012 ▸) that the two tiling spaces generated by T–S tilings and Penrose tilings define distinct MLD classes, it is clear by now that the two types of tilings are intimately related, and indeed, modulo the choice of a coset, there is a mutual derivability. We can summarize some key points as follows:

**Theorem 7.1**

(i) Taylor–Socolar tilings and Penrose tilings are aperiodic.

(ii) Given a non-singular Taylor–Socolar tiling on *Q*, one can build, in a canonical way, three different non-singular Penrose tilings, one for each of the three cosets 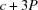 of 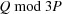. At any point of 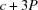 one knows exactly what type of Penrose tile should be put in that position, and this uses only local information of the T–S tiling.

(iii) Given a non-singular Penrose tiling on some coset 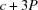, there is a unique nested triangulation on *Q* formed by the decoration of inner hexagons and corner hexagons of Penrose tiles. This nested triangulation gives a unique non-singular T–S tiling. Note that, unlike the situation in (ii), this construction is not local.

The process of producing double hexagon tiles from a pair 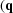, 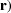 suggests that we might do it again, choosing a coset 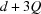 with 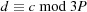 and then determining 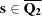. This triple 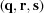 leads to triple hexagon tiles and a triple hexagon tiling. The rules for admissibility follow the same principles as we have used above. The largest hexagonal tiles have middle-sized hexagonal tiles at their centers, and create middle-sized corner tiles around them. The requirement is well arrowing throughout. This yields a well arrowed hexagonal tiling of middle-sized tiles. In the same way, we can create from this a hexagonal tiling of small tiles, where again we require well arrowing throughout.

**Figure 22:**
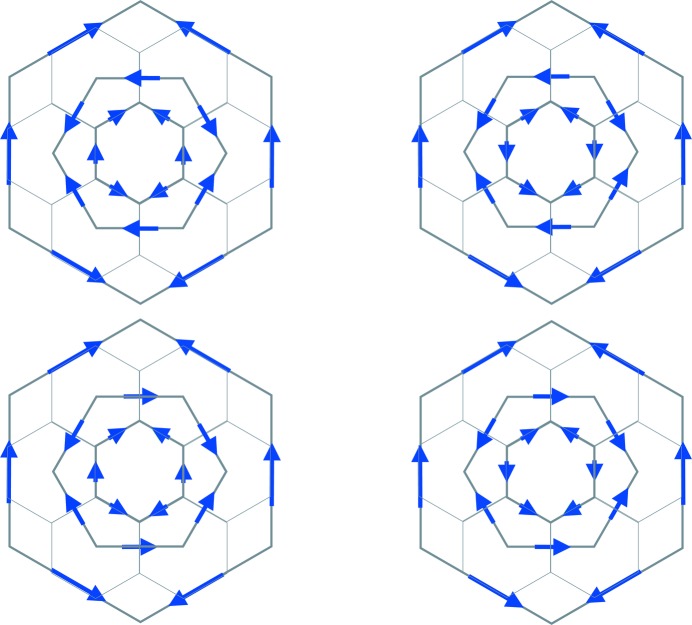
The four types of triple (or 3-tiered) hexagons.

These triple tiles come in four types and produce a new type of hexagonal tiling (Fig. 22 ▸). There is no reason to stop there. This new hierarchical situation is illustrated in the commutative diagram (3):
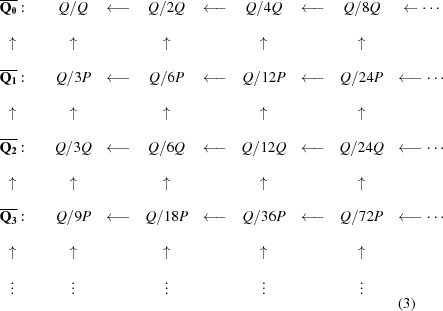



More generally there are ‘*n*-tuple hexagons’, or to have a better sounding name, *n*-*tiered hexagons*, each of which consists of *n* hexagons stacked within each other, which tile the plane according to the *n*th line of (3). There are two choices for the orientation of a hexagon at each stage of layering, but after taking into account rotations, this gives 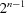 types of these tiered tiles. Non-singularity (respectively, singularity) is a property common to all levels.

## 8. Outlook   

The purpose of this paper has been to clarify the unity that exists between the Taylor–Socolar tilings and the Penrose hexagonal tilings – a unity that can be expressed both geometrically and algebraically in terms of double hexagon tiles. Each non-singular legal double hexagon tiling encompasses both a Penrose tiling and a T–S tiling, and this pairing can be interpreted algebraically in terms of (2). Each of the two hexagonal tilings leads to a nested triangulation, and these two are bound together by the simple rule that triangle edges of each right-bisect edges of the other.

There are two issues that arise here that we have not discussed, but plan to pursue in future work. The first is the nature of singularities in these tilings from both the geometric and algebraic perspectives, and their detailed manifestation in the corresponding tiling hulls. The second is the study of the *n*-tiered hexagonal tilings. The algebraic setting which uses the first *n* rows of the commutative diagram (3) suggests that the *n*-tiered hexagons lead to aperiodic tilings in which there are potentially 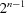 types of tiles. Thus there is a hierarchy of aperiodic hexagonal tilings, and their corresponding tiling hulls, about which we know very little.

## Footnotes

1 Since Penrose tilings based on fivefold symmetry are so much a part of the aperiodic culture, we should emphasize that the Penrose tilings of this paper are based on hexagons and have nothing to do with the rhombic or kite/dart Penrose tilings.
